# “Snake flu,” “killer bug,” and “Chinese virus”: A corpus-assisted critical discourse analysis of lexical choices in early UK press coverage of the COVID-19 pandemic

**DOI:** 10.3389/frai.2022.970972

**Published:** 2022-11-22

**Authors:** Ursula Kania

**Affiliations:** Department of English, University of Liverpool, Liverpool, United Kingdom

**Keywords:** corpus-assisted discourse studies, corpus linguistics, critical discourse analysis, lexical choices, Sinophobia, Anti-Asian racism, UK press, COVID-19

## Abstract

Now mostly known as “COVID-19” (or simply “Covid”), early discourse around the pandemic was characterized by a particularly large variation in naming choices (ranging from “new coronavirus” and “new respiratory disease” to “killer bug” and the racist term “Chinese virus”). The current study is situated within corpus-assisted discourse studies and analyses these naming choices in UK newspaper coverage (January–March 2020), focusing on terminology deemed “inappropriate” as per WHO guidelines on naming infectious diseases. The results show that 9% of all terms referring to COVID-19 or the virus causing it are “inappropriate” overall, with “inappropriate” naming being more prevalent (1) in tabloids than broadsheets and (2) in the period before compared to the period after the virus was officially named on 11th February, 2020. Selected examples within each of the categories of “inappropriate” names are explored in more detail [terms (1) inciting undue fear, (2) containing geographic locations, and (3) containing species of animals], and the findings are discussed with regard to the contribution of lexical choices to the reproduction of (racist and otherwise problematic) ideologies in mainstream media.

## Introduction

The first cases of the disease that would become known as COVID-19 were identified in central China in December 2019, and media coverage in early 2020 often linked the outbreak specifically to the Huanan Seafood and Wildlife Market in Wuhan. Since then, the spread of COVID-19 has been accompanied by a rise in Anti-Asian hate speech and hate crime in many countries (for the US, see Gover et al., 2020; for the UK, see Gray and Hansen, 2021). It has already been noted that “[t]hroughout history, pandemic-related health crises have been associated with the stigmatization and “othering” of people of Asian descent” (Gover et al., 2020, p. 647). This “othering” has often involved the conflation of different ethnicities (e.g., viewing all “Asians” as a monolithic group; Yeh, 2020) and perpetuation of pernicious stereotypes, for example of (alleged) Chinese foodways as “exotic” or “disgusting” and potentially to blame for the spread of diseases (King, 2020).

In light of this history, terms such as “Chinese virus” or “Wuhan virus” are highly problematic and inappropriate, since they further contribute to a construal inextricably linking the virus and the illness it causes to China. They also do not comply with WHO guidelines (WHO, 2015), which aim to minimize negative effects potentially resulting from inappropriate naming. The current study focuses on lexical choices around COVID-19 and Sars-CoV-2 in one specific context, i.e., UK press coverage from January until March 2020, aiming to provide a critical analysis of newspapers' “politics of naming” from the perspective of corpus-assisted discourse studies.

## Background and previous research

The WHO guidelines for “Best practices for the Naming of New Human infectious diseases” state that disease names should be carefully chosen to “avoid causing offense to any cultural, social, national, regional, professional, or ethnic groups” (WHO, 2015, p. 1). The guidance is designed to “span the gap between identification of a new human disease event and assigning a final name by ICD [International Classification of Diseases]” (ibid.), offering “examples of useful terms” as well as “examples to be avoided,” the latter of which include “terms that incite undue fear” (such as “death” or “fatal”), “geographic locations,” “people's names,” “species/class of animals or food” (ibid., p. 3).^1^ While these guidelines cover diseases specifically (not the pathogens causing them), the organization responsible for naming viruses—the International Committee on Taxonomy of Viruses (ICTV) is also aware of potentially harmful consequences and follows a code according to which “[n]ew names shall be chosen with due regard to national and/or local sensitivities” (ICTV, 2021). Furthermore, the WHO states that “WHO and ICTV were in communication about the naming of both the virus and the disease” (WHO, 2020). Consequently, the official names, announced on 11th February 2020, do not include any terms deemed inappropriate: coronavirus disease (or COVID-19), caused by severe acute respiratory syndrome coronavirus 2 (or SARS-CoV-2; replacing the temporary name “2019-nCoV,” which was assigned on January 7th, 2020) (ibid.). However, the WHO states that “using the name SARS can have unintended consequences in terms of creating unnecessary fear for some populations, especially in Asia which was worst affected by the SARS outbreak in 2003,” therefore they are “referring to the virus as “the virus responsible for COVID-19” or “the COVID-19 virus” when communicating with the public” (ibid.; for a critical discussion of the naming of the virus, see Jiang et al., 2020).

It is thus evident that lexical choices (not only) pertaining to the illness and the virus causing it matter, with inappropriate terms potentially exacerbating pre-existing stereotypes, discrimination, and racism (for “Reflections on the Racialised Discourse surrounding COVID-19,” see Ng et al., 2021, pp. 144–146; also see Wang et al., 2021, for a broader discussion of “Representations of “China” in Britain”)^2^. Some evidence for this connection has already been provided. For example, tweets including the hashtag #chinesevirus have been found to be much more likely to express Anti-Asian sentiment compared to more neutral ones such as #covid19 (Hswen et al., 2021). For the US, it has also been shown that a preference for a particular framing in the media (use of “COVID-19 virus” vs. “Chinese virus”) aligns with people's political affiliation/ideology (Democrat/Republican and liberal/conservative), and that “amongst a host of other variables, media framing has an effect on the public's attitudes and feelings of blame for the pandemic” (Holt et al., 2022).

The study most directly related to the current one is Prieto-Ramos et al. (2020), who analyze relevant naming choices in the headlines of 2 newspapers each for the US, the UK, France, and Spain (in January and February 2020). They found a drastic reduction of inappropriate naming in all newspapers after the WHO announcement. For the two UK broadsheet newspapers they included (*The Times* and *The Telegraph*), inappropriate terms were found in 8.63 and 5.56% of all headlines “pre-naming,” respectively, and in none at all “post-naming.” Even though they briefly discuss the controversy around Donald Trump's use of “Chinese virus,” there is no in-depth analysis, since their dataset does not extend to March 2020 when Trump used this term repeatedly.

While also being concerned with these “politics of naming,” the current study has a different scope and focus: it deals exclusively with the UK context but includes more newspapers, which allows for a comparison between tabloid vs. broadsheet publications. Furthermore, the time-frame is slightly longer (extending to 31st March 2020), providing more data “post-naming” and making it possible to observe longitudinal shifts in reporting (as well as coverage of Trump's use of “Chinese virus”). Lastly, while more specific search terms were used for the compilation of the current corpus (see methods section below), it includes the full text of articles, not just the main headlines, making it possible to analyse the broader context from a discourse analytic perspective as well.

## Methods

This corpus-based study is situated within corpus-assisted discourse studies (henceforth CADS; see e.g., Partington, 2004; Partington et al., 2013; Ancarno, 2020) and thus combines corpus linguistics and discourse analysis. The approach has been chosen because of “CADS's” ability to reconcile close linguistic analyses with the more broad-ranging analyses made possible by using corpus linguistic methods […], [which] allows for insights into micro- and macro-level phenomena to be explored simultaneously” (Ancarno, 2020, p. 165).

The contribution made by corpus linguistics methods consists of the compilation of a specialized corpus, the analysis of absolute and relative frequencies of relevant terms, the identification of collocates for the two most frequent head nouns, and the use of selected concordances for explorations of their discourse context (using *AntConc*; Anthony, 2020). Corpus linguistic techniques are combined with a close reading approach from the perspective of critical discourse analysis, drawing on the notion of ideology as well as previous research on newspaper language and lexical choices therein.

As ““systems of ideas,” ideologies are sociocognitively defined as shared representations of social groups […] [I]deologies organize [a social group's] identity, actions, aims, norms and values, and resources as well as its relations to other social groups” (van Dijk, 2006, p. 115). Since they “are acquired, expressed, enacted and reproduced by discourse, this must happen through a number of discursive structures and strategies” (ibid., p. 126). In particular, “ideologies are institutionally co-produced and reproduced by powerful (business) institutions such as newspapers” (ibid., p. 138), so their discursive strategies are of primary interest. The idea that newspaper language is far from “neutral” is not new (see, e.g., Kress, 1983), and since the “variation of lexical items (that is, lexical style) is a major means of ideological expression in discourse” (van Dijk, 2000, p. 205), lexical choices often receive analytical attention (e.g., van Dijk, 1988, 1991, 1995; Crespo Fernández and Martínez Lirola, 2012 of course, other semiotic systems such as images are also important; see, e.g., Machin, 2013).

Apart from the choices *per se*, it is crucial to consider how they are embedded within articles, e.g., through various means of speech representation (see, e.g., the framework proposed by Semino and Short, 2004; one study applying it to UK newspaper data is Lampropoulou, 2014). This means that a decontextualized, quantitative analysis of specific lexical items is just the first step, which has to be followed by an in-depth look at the broader discourse context.

## Data

Data for this study consist of the COVID-19-related corpus collected for a research project on Sinophobia and representations of Chinese (food) culture in the UK press (focusing on historical and COVID-19-related manifestations; see Kania and González-Díaz, in preparation). For 1st January until 31st March, 2020, i.e., the early stages of the COVID-19 pandemic, relevant data were extracted from *Nexis*[search string used: (Covid^*^ OR corona^*^ OR “SARS-CoV-2” OR virus OR ^*^nCoV^*^) AND (Chine^*^ OR China^*^) AND (food^*^ OR eat^*^ OR consum^*^ OR cook^*^ OR restaurant^*^ OR takeaway^*^)]. Consequently, not all UK news articles covering COVID-19 from January until March 2020 are included here but only those mentioning China (and foodways) in some way.^3^ The corpus consists of 555 articles from both tabloid and broadsheet publications, including online versions (where available), totaling 716,411 words. An overview of the composition of the corpus is presented in Table 1.

**Table 1:** Overview of the corpus composition.

**Tabloid**	**Print circulation (Mayhew, 2020)**	**Online**	**Articles**	**Words**
The Daily Mail/Mail on Sunday	1,169,241/967,043	X	188	338158
The Metro	1,426,535	–	2	435
The Mirror/Sunday Mirror	451,466/367,244	X	51	32874
The Sun/The Sun on Sunday	1,250,634/1,042,193	X	47	34885
The Daily Star/The Daily Star Sunday	277,237/162,345	X	25	11749
The Express/Sunday Express	296,079/252,733	X	32	24191
		Total	345	442292
**Broadsheet**	**Print circulation**	**Online**	**Articles**	**Words**
The Guardian/Observer	132,341/156,217	X	70	162854
The Independent (published online only)	n/a	X	49	30163
The Times/Sunday Times	368,929/645,108	X	50	41236
Telegraph	Not available	X	40	37923
		Total	210	274119

This study is mainly interested in the distribution of different “neutral” vs. “inappropriate” terms for COVID-19 and the associated pathogen in the time-frames before and after the official names were announced. It is also interested in differences between broadsheet and tabloid coverage, both in terms of absolute and relative frequencies of “inappropriate” terms and how “inappropriate” terms such as “Chinese virus” are embedded in the articles and how they contribute to the construction and reproduction of particular ideologies.

## Results and discussion

As stated above, the official names were only announced on 11th February 2020, so different lexical choices were available before and after. Therefore, following the approach by Prieto-Ramos et al. (2020), the dataset has been split into two timeframes: (1) 1st January–10th February (41 days, pre-naming, average number of news stories per day = 7.24), and (2) 11th February–31st March (50 days, post-naming, average number of news stories per day = 5.16). An overview of the subsets can be found in Table 2. Overall, there are more articles in the pre-naming than in the post-naming timeframe (297 vs. 258), despite the former being shorter, potentially because some later coverage may not have mentioned China (instead focusing on UK-specific information on the first lockdown, for example). Furthermore, there was a decrease in the tendency, particularly by tabloids, to publish several online news stories per day—there are fewer tabloid articles post-naming (199 vs. 146), while there is actually a slight increase in broadsheet coverage (98 vs. 112).

**Table 2:** Corpus composition, broken down by tabloid vs. broadsheet and “pre-naming” vs. “post-naming.”

**Pre-naming**	**Articles**	**Words**	**Post-naming**	**Articles**	**Words**
Broadsheet	98	129190		112	144929
Tabloid	199	262162		146	180190
Totals	297	391352		258	325199

Exploratory searches were done for likely lexical choices (e.g. “^*^virus” and “illness”), and further terms were identified by close reading of all headlines and a random sample of 100 articles in the dataset (25 each for tabloid and broadsheet pre- and post-naming). References to other illnesses and viruses (e.g., SARS and Zika) were identified and excluded manually through the inspection of all concordance lines. For this analysis, context for key head nouns included in the table was limited to pre-modifiers. Cases where the noun for the virus or illness was used as the first part of a compound (e.g., “coronavirus outbreak”) were included here as well (e.g., under “coronavirus”), unless the relevant compound containing a term for the virus denoted the “illness”, in which case it was included in the counts for the illness (e.g., “new viral coronavirus illness”).

### “Neutral” vs. “inappropriate” terms

The first analyses on lexical choices focus on absolute and relative frequencies of different terms used for (1) the virus officially called SARS-CoV-2 and (2) the illness it causes, officially named COVID-19. While in theory there is a clear distinction between terms for the virus and the illness, respectively, in practice the boundaries are often blurred, with e.g., COVID-19 being used for the virus (e.g., “The new virus, officially called Covid-19,” *The Telegraph*, 19th March, 2020) or a term for the illness being used as a synonym for the virus (“Wuhan pneumonia is the name for a new coronavirus,” *Daily Mirror Online*, 24th January, 2020). This is why no strict boundary between these two categories was imposed in the presentation of the results.

The guidelines do not explicitly state that comparisons to similar pathogens should be avoided (e.g., “SARS-like virus”)—however, the WHO ultimately recommended to avoid the term SARS, since it may “create unnecessary fear” (WHO, 2020; see discussion above) and it has thus been categorized as “inappropriate.” Furthermore, “unknown” is explicitly listed by the WHO as an example to be avoided, so similar terms such as “mysterious” and “previously unknown” were also categorized as inappropriate. In other cases, though, a fairly conservative approach was taken—for example, “highly-contagious,” while potentially inducing fear, was deemed appropriate since “contagious” is included in the WHO examples of “useful terms.”

Since the focus here is on neutral vs. “inappropriate” lexical choices, counts for terms within these categories have been conflated for each of the head nouns for the presentation of the results in Table 3 (the head nouns are: virus, coronavirus, bug, corona, n-CoV, SARS-CoV-2, COVID-19, condition, flu, plague, infection, disease, illness, pneumonia).

**Table 3:** Overview of frequencies of “neutral” vs. “inappropriate” terms (“inappropriate” terms and counts in italics, total counts per term and overall counts in bold).

	**Broadsheet 01/01-10/02**	**Broadsheet 11/02-31/03**	**Tabloid 01/01-10/02**	**Tabloid 11/02-31/03**	**Total**
Total virus “neutral”	383 (4)	931 (4)	1848 (5)	734 (8)	**3896 (21)**
*Total virus “inappropriate”*	*54 (4)*	*32 (3)*	*258 (24)*	*65 (1)*	***409 (32)***
Total virus	**437 (8)**	**963 (7)**	**2106 (29)**	**799 (9)**	**4305 (53)**
Total coronavirus “neutral”	673 (35)	739 (59)	1653 (93)	1302 (83)	**4367 (270)**
*Total* coronavirus “*inappropriate”*	*52 (7)*	*4 (–)*	*339 (13)*	*28 (–)*	***423 (20)***
Total coronavirus	**725 (42)**	**743 (59)**	**1992 (106)**	**1330 (83)**	**4790 (290)**
Total bug “neutral”	1 (1)	3 (–)	14 (–)	14 (1)	**32 (2)**
*Total bug “inappropriate”*	–	–	*15 (6)*	*16 (2)*	***31 (8)***
Total bug	**1 (1)**	**3 (–)**	**29 (6)**	**30 (3)**	**63 (10)**
Total nCoV “neutral”	23 (–)	1 (–)	82 (–)	9 (–)	**115 (–)**
*Total nCoV “inappropriate”*	–	–	–	–	***–***
Total nCoV	**23 (–)**	**1 (–)**	**82 (–)**	**9 (–)**	**115 (–)**
Total SARS-CoV-2 “neutral”	–	6 (–)	–	10 (–)	**16 (–)**
*Total SARS-CoV-2 “inappropriate”*	–	–	–	*1 (–)*	***1 (–)***
Total SARS-CoV-2	**–**	**6 (–)**	**–**	**11 (–)**	**17 (–)**
Total COVID(-19) “neutral”	–	219 (5)	–	272 (–)	**491 (5)**
*Total COVID-19 “inappropriate”*	–	–	–	*3 (–)*	***2 (–)***
Total COVID(-19)	**–**	**219 (5)**	**–**	**275 (–)**	**494 (5)**
Total condition “neutral”	1 (–)	–	14 (–)	–	**15 (–)**
*Total condition “inappropriate”*	–	–	*7 (–)*	–	***7 (–)***
Total condition	**1 (–)**	**–**	**21 (–)**	**–**	**22 (–)**
Total flu “neutral”	2 (2)	– (–)	– (–)	–	**2 (–)**
*Total flu “inappropriate”*	*7 (–)*	*3 (–)*	*29 (1)*	*6 (–)*	***45 (1)***
Total flu	**9 (2)**	**3 (–)**	**29 (1)**	**6 (–)**	**47 (3)**
Total plague “neutral”	–	–	–	–	**–**
*Total plague “inappropriate”*	–* (–)*	*3 (–)*	*3 (2)*	*2 (–)*	***8 (2)***
Total plague	**– (–)**	**3 (–)**	**3 (2)**	**2 (–)**	**8 (2)**
Total infection “neutral”	81 (1)	57 (1)	140 (1)	140 (–)	**418 (3)**
*Total infection “inappropriate”*	*5 (–)*	–* (–)*	*47 (1)*	*9 (–)*	***61 (1)***
Total infection	**86 (1)**	**57 (1)**	**187 (2)**	**149 (–)**	**479 (4)**
Total disease “neutral”	108 (–)	89 (1)	203 (2)	93 (–)	**493 (3)**
*Total disease “inappropriate”*	*6 (1)*	–* (–)*	*32 (4)*	*9 (1)*	***47 (6)***
Total disease	**114 (1)**	**89 (1)**	**235 (6)**	**102 (1)**	**540 (9)**
Total illness “neutral”	32 (–)	14 (–)	107 (1)	59 (–)	**212 (1)**
*Total illness “inappropriate”*	*14 (2)*	*1 (–)*	*14 (–)*	*3 (1)*	***32 (3)***
Total illness	**46 (2)**	**15 (–)**	**121 (1)**	**62 (1)**	**244 (4)**
Total pneumonia “neutral”	33 (1)	9 (–)	224 (2)	4 (–)	**270 (3)**
*Total pneumonia “inappropriate”*	*8 (1)*	*1 (–)*	*3 (–)*	*1 (–)*	***13 (1)***
Total pneumonia	**41 (2)**	**10 (–)**	**227 (2)**	**5 (–)**	**283 (4)**
Overall total “neutral”	**1337 (44)**	**2068 (70)**	**4285 (104)**	**2637 (92)**	**10327 (310)**
*Overall total “inappropriate”*	*146 (15)*	*44 (3)*	*747 (51)*	*143 (5)*	*1080 (74)*
Overall total	**1483 (59)**	**2112 (73)**	**5034 (155)**	**2780 (97)**	**11407 (384)**

A full list of terms and the breakdown of their frequencies is made available as Supplementary Table 1.

Furthermore, selected terms will be discussed in more detail below.

The first number in each cell provides the total count for the category, whereas the number in brackets indicates how many of the instances were included in a main headline.

Overall, there are 11,407 explicit mentions of either COVID-19 or the virus causing it in the whole corpus-−1,080 (or 9%) of these terms have been categorized as “inappropriate” (percentages are rounded to the nearest whole number). Inappropriate terms are particularly prevalent in headlines (74 out of 384, i.e., 19%). The vast majority of “inappropriate” terms are found “pre-naming” (895 out of 6,515, i.e., 14%) rather than “post-naming” (174 out of 4,892, i.e., 4%), and the same trend can be observed for headlines (66 out of 214, i.e., 31% for “pre-naming” as opposed to 8 out of 170, i.e., 5% for “post-naming”).

This indicates a shift toward more “neutral” terminology over time, with the terms “virus,” “coronavirus” and the official name “COVID-19” being the most frequent choices (“SARS-CoV-2” as the official name for the virus is only used 17 times and—with only 5 uses—“Covid” is not an established term yet). This shift is broadly in line with Prieto-Ramos et al. (2020), who found that “inappropriate names were dramatically reduced in the news headlines of the mainstream media observed” (p. 464)—however, with 8.63% (*The Times*) and 5.56% (*The Telegraph*) “pre-naming,” and no inappropriate headlines at all “post-naming,” the prevalence of “inappropriate” headlines is less pronounced in their dataset. This might be due to differences in criteria for data selection: while they were more general in their search terms (as opposed to including only coverage mentioning China and associated foodways alongside COVID-19), they only included two UK broadsheet newspapers (and no tabloids at all), and their “post-naming” was limited to 12–29th February 2020 (i.e., not extending until 31st March like in the current study).

Regarding the choice of newspapers: in the current dataset, 1 out of 14 relevant headlines in *The Times* (i.e., 7%) and 3 out of 19 relevant headlines in *The Telegraph* (i.e., 16%) are “inappropriate” “pre-naming,” and they contribute none of the 3 inappropriate broadsheet headlines “post-naming,” which aligns with Prieto-Ramos et al.'s results for these publications overall. So while the search terms may have had some influence, the differences are probably mostly driven by the other newspapers included. Since we may expect tabloids to make more use of sensationalist language (see, e.g., Wahl-Jorgensen, 2020), this aspect will be evaluated first.

For broadsheets, 146 out of 1,483 (i.e., 10%) terms overall are “inappropriate” “pre-naming” and 44 out of 2,112 “post-naming” (i.e., 2%), whereas for tabloids it is 747 out of 5,032 “pre-naming” (i.e., 15%) and 143 out of 2,780 “post-naming” (i.e., 5%).

For headlines only, “inappropriate” terms are included in 15 out of 59 for broadsheets “pre-naming” (i.e., 25%) and 3 out of 73 “post-naming” (i.e., 4%), whereas for tabloids it is 51 out of 155 headlines “pre-naming” (i.e., 33%) and 5 out of 97 “post-naming” (i.e., 5%).

This means tabloids do drive the numbers up, but since the percentage for inappropriate headlines in broadsheets is still higher than indicated by Prieto-Ramos et al. (2020), this indicates that the broadsheet newspapers *The Guardian* and *The Independent* have a stronger tendency to include inappropriate terms in their headlines compared to *The Times* and *The Telegraph* (since for broadsheets the overall percentage of inappropriate headlines pre-naming is 25%). In sum, it is likely that there are multiple factors at play here but the main cause seems to lie in the stronger tendency of the additional broadsheet and tabloid newspapers considered here to use “inappropriate” terms.

Overall, “inappropriate” terms constitute about 9% of all uses—they are more frequent in the “pre-naming” vs. the “post-naming” period, and—except for tabloids “post-naming”—particularly prevalent in main headlines. Throughout, broadsheets have a lower absolute and relative frequency of “inappropriate” terms compared to tabloids.

To get a first impression of which pre-modifiers are particularly prevalent in a corpus-linguistic sense, the top 20 3L-collocates were identified for the two most frequent head nouns (“coronavirus”, *n* = 4,790, “virus,” *n* = 4,305; see Supplementary Tables 2, 3 for parameters and full results).

For “coronavirus,” “novel” features as one of the “appropriate” pre-modifiers throughout all sub-corpora (i.e., broadsheet as well as tabloid, pre- as well as post-naming). The most consistently used “inappropriate” pre-modifier is “deadly” (broadsheet pre- and post-naming and tabloid pre-naming), with the even stronger expression “killer” only reaching statistical significance in tabloids (both pre- and post-naming). For “virus,” on the other hand, there is no “appropriate” pre-modifier/determiner found throughout (for broadsheet, there is “new” and the pre-naming and “the/this” as well as SARS-CoV post-naming; for tabloids, there are no relevant collocates in the top 20 at all). Similar to “coronavirus,” “deadly” features as one of the “inappropriate” pre-modifiers (except for tabloids pre-naming). Interestingly, “killer” is not only found in tabloids (pre- and post-naming), but also in broadsheets pre-naming, and “Chinese” is found only for broadsheets (post-naming), indicating that specific “inappropriate” uses may in fact be more predominant in broadsheets rather than tabloids.

### Use of “inappropriate” terms

Since an exhaustive analysis of all “inappropriate” terms is beyond the scope of this paper, the focus is on selected examples within these categories: (1) Terms inciting undue fear, (2) Terms including geographic locations, and (3) Terms including the names of species of animals, in each case starting with overall frequencies before analyzing selected examples in context.

#### Terms inciting undue fear

For broadsheets, 70 out of 146 (i.e., 48%) “inappropriate” terms “pre-naming” contain expressions inciting undue fear, as opposed to 14 out of 44 (i.e., 32%) “post-naming”. For tabloids, it is 504 out of 747 “pre-naming” (i.e., 67%) and 126 out of 143 (i.e., 88%) “post-naming.”^4^

For headlines only, it is 11 out of 15 for broadsheets “pre-naming” (i.e., 73%) and 0 out of 3 “post-naming” (i.e., 0%), whereas for tabloids it is 45 out of 51 “pre-naming” (i.e., 88%) and 5 out of 5 “post-naming” (i.e., 100%). This means that terms inciting undue fear are present in both broadsheets and tabloids but—both in absolute and relative terms—tabloids make more use of terms like “deadly coronavirus,” particularly in headlines. Furthermore, while both broadsheets and tabloids make use of the pre-modifiers “deadly” or “mysterious,” tabloids are more likely to use particularly sensationalist terms such as “killer bug” or “killer virus” (the latter of which is used 51 times “pre-naming” and 8 times “post-naming” by tabloids, and occurs in 5 headlines “pre-naming”)—in fact, the only term containing “killer” found in broadsheets is “killer virus.” While this is used 6 times, an analysis of concordance lines reveals that all uses are quotes and refer to coverage in other media outlets such as the tabloid *The Daily Mail*:

(1) “Is the **killer virus** here?” shrieks the headline on the Daily Mail (emphasis added; *The Guardian*, 23th January, 2020).

This is not the only example of explicit intertextuality, with broadsheets quoting or referring to tabloid coverage, usually in the context of a negative evaluation (see the discussion of “snake flu” below).

#### Terms including geographic locations

For broadsheets, 69 out of 146 (i.e., 47%) “inappropriate” terms “pre-naming” contain a geographic location, as opposed to 25 out of 44 (i.e., 57%) “post-naming.” For tabloids, it is 249 out of 747 “pre-naming” (i.e., 33%) and 15 out of 143 (i.e., 10%) “post-naming.” For headlines only, it is 9 out of 15 for broadsheets “pre-naming” (i.e., 60%) and 3 out of 3 “post-naming” (i.e., 100%), whereas for tabloids it is 13 out of 51 “pre-naming” (i.e., 25%) and 0 out of 5 “post-naming” (i.e., 0%). This means that, in relative terms, this inappropriate naming strategy is more prevalent in broadsheet vs. tabloid newspapers, in part driven by the stronger tendency of the latter to include terms inciting undue fear, as discussed above. It might also indicate, though, that the inclusion of terms such as “Wuhan,” “China,” or “Chinese” is seen as relatively unproblematic, particularly by broadsheet newspapers, for which the relative use even increases “post-naming” compared to “pre-naming.”

A closer look at the distribution of terms shows that the vast majority of cases within this category refer to SARS-CoV-2 as “Wuhan (corona)virus” or “Chinese (corona)virus,” sometimes with additional pre-modifiers like “new,” “deadly,” or “killer,” with other terms such as “mystery China disease” or “deadly China virus” only appearing rarely. The locally more specific “Wuhan (corona)virus” dominates “pre-naming” for both broadsheets [with 52 vs. only 4 instances of “Chinese (corona)virus”] and tabloids [with 161 vs. 61 instances of “Chinese (corona)virus”]. It all but disappears “post-naming” (with no uses in broadsheets and only 7 instances in tabloids). While Prieto-Ramos et al. (2020, p. 646) view “Wuhan” as less inappropriate than “Chinese,” since the latter “represents a broader generalization,” some coverage clearly construes the “Wuhan coronavirus” as being linked to China more generally:

(2) An infected doctor in France became the country's first person to catch the **killer Wuhan coronavirus** without going to China (emphasis added; *Daily Mail Online*, 31st January, 2020).

It should be stressed, though, that some experts which are quoted in the news coverage use the term Wuhan as well, so in these cases the naming practices may be argued to reflect “the information available to public authorities and journalists during the first period of unstable naming” (Prieto-Ramos et al., 2020, p. 646; note that this makes the case for educating professionals on appropriate language even stronger—see e.g., Vazquez, 2020):

(3) “I think it unlikely that the **Wuhan coronavirus** will cause a major public health issue in the UK, in large part because of our existing health system.” (emphasis added; *The Guardian*, 23rd January, 2020—featured quote by Paul Hunter, professor in medicine at the University of East Anglia).

As stated above, Chinese (corona)virus is less prevalent than Wuhan (corona)virus pre-naming and in contrast to the latter there is already some awareness and explicit coverage (though only in broadsheets) of the term being potentially problematic:

(4) Raymond Huo, a local MP, said the coronavirus matter was the “number one issue” in the Chinese community. “We are concerned about any racist comments or discriminatory behavior. There have been a few isolated cases,” he said, adding that negative sentiment and fear had been fuelled by headlines describing the disease as a “Chinese virus.” *(The Telegraph*, 1st February, 2020).

**Figure 1:**
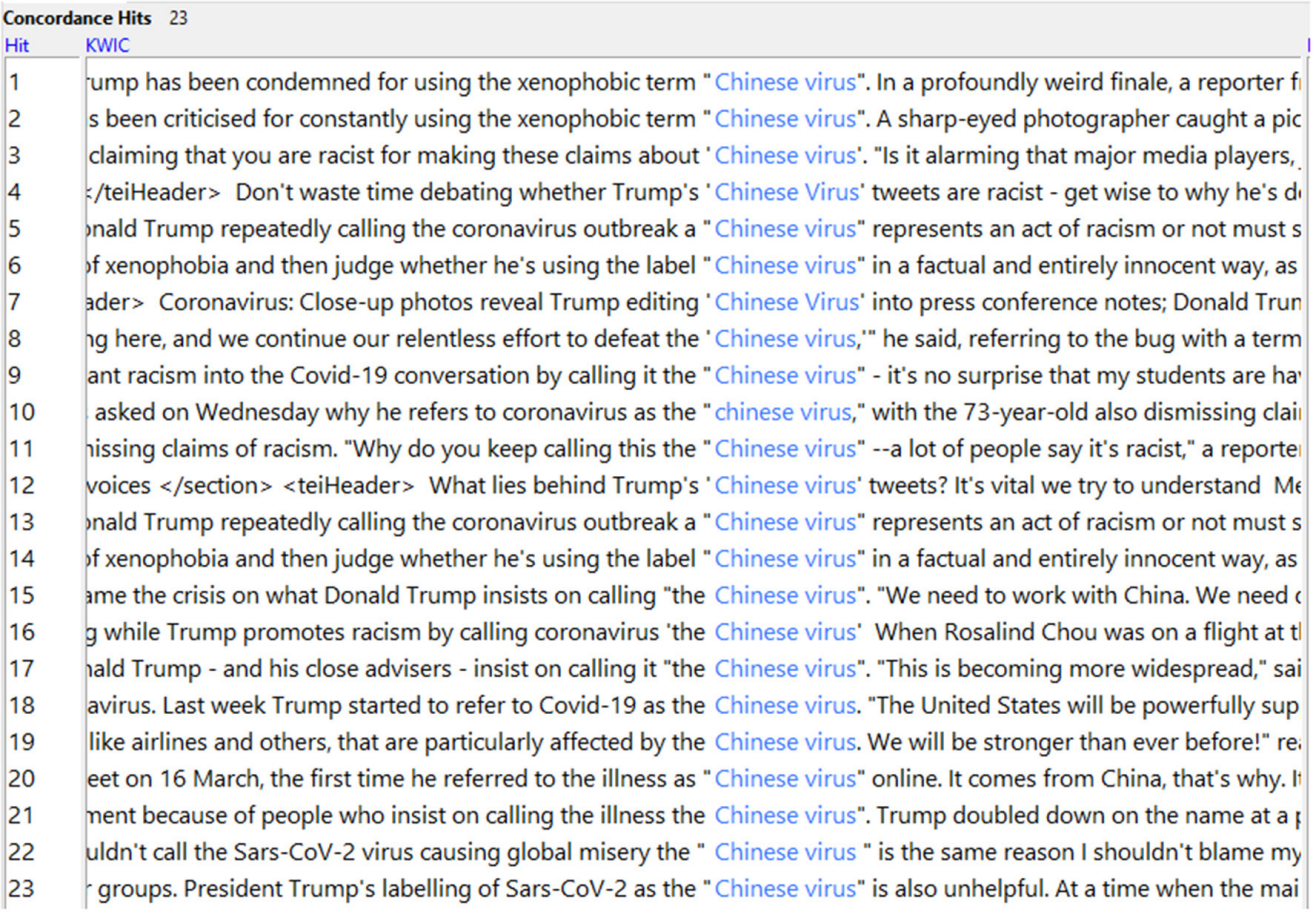
Concordance lines for “Chinese virus” in broadsheets (“post-naming”).

It is quite striking, then, that the use of “Chinese (corona)virus” increases in broadsheet coverage “post-naming” (from 4 to 24 uses—for tabloids, there is a decrease from 61 to 5 uses). Again, a close look at the concordance line reveals that decontextualized frequency data does not tell the whole story—all 23 uses of “Chinese virus” in broadsheets are construed as (parts of) quotes, predominantly linking it to then-US president Donald Trump (see Figure 1).

This ties in with the overall stance taken by broadsheets, which—particularly “post-naming”—attribute problematic terms to other people (or media outlets) and provide an explicit negative evaluation of these lexical choices (e.g., referring to the term “foreign virus” as “xenophobic rebranding by Donald Trump”; *The Guardian*, 13th March, 2020).

**Figure 2:**
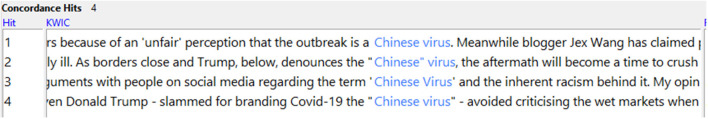
Concordance lines for “Chinese virus” in tabloids (“post-naming”).

This is in contrast to the dominant construal found in tabloids—“pre-naming,” choice of terminology is usually not problematized, and even though there are way fewer instances of inappropriate terms post-naming (see Figure 2 for concordance lines of “Chinese virus”), there is a tendency to present a negative evaluation of using problematic terms to individuals featured in the article rather than the stance of the newspaper itself (see example 5).

(5) Parents have claimed Chinese children are being ostracized by their friends in British schools, with some refusing to play with them. Mothers have told the BBC that people are being **“racist”** against the youngsters because of an **“unfair”** perception that the outbreak is a **Chinese virus** (emphasis added, *The Daily Mail Online*, 14th February, 2020).

Furthermore, there is a piece entitled “Let's get angrier at cruel markets that caused virus,” which implies that Donald Trump does not go far enough in his assignment of blame for COVID-19:

(6) So why is there so little outrage about the wet markets that we know have the potential to cause catastrophic outcomes to human health? Even Donald Trump—slammed for branding COVID-19 the **“Chinese virus”**—avoided criticizing the wet markets when prompted during a press conference at the White House on Wednesday (emphasis added, *The Sun*, 27th March, 2020).

#### Terms including the names of species of animals

For broadsheets, 5 out of 146 (i.e., 3%) “inappropriate” terms “pre-naming” contain animal names, as opposed to 4 out of 44 (i.e., 9%) “post-naming.” For tabloids, it is 32 out of 747 “pre-naming” (i.e., 4%) and 6 out of 143 (i.e., 4%) “post-naming.”

Only 4 instances occur in headlines (all for tabloids “pre-naming”). This is the only category that not featuring in Prieto-Ramos et al. (2020), since there are no occurrences in the headlines of *The Times* or *The Telegraph*. The predominant term is “(deadly) (Chinese) snake flu”—used by broadsheets 3 times each “pre-” and “post-naming” and 27 times “pre”- and 6 times “post-naming” by tabloids.

It first appears in *The Daily Mirror*, where its potential impact is compared to other diseases such as the “Marburg virus” or “Lassa fever”:

(7) **Snake flu**, as it will surely become known, could turn out to be worse than all of those (emphasis added, *Daily Mirror Online*, 24th January, 2020).

Like observed for “killer bug” above, all the mentions in broadsheets do, in fact, refer to tabloid coverage, and even though there are way fewer mentions in tabloids “post-naming” (and none at all after 2nd March, 2020), this lexical choice is salient enough to be explicitly commented on:

(8) [O]ne tabloid [is] seemingly desperate for the moniker **“snake flu”** to catch on, because snake flu sounds so much slicker and scarier than boring old COVID-19, doesn't it? Who the hell do these people from the WHO think they are, trying to be responsible with the naming of this illness so as not to create stigma? What do we want? **Snake flu**! When do we want it? NOW! (emphasis added, *The Telegraph*, 15th February, 2020).

The misnomer is particularly relevant for a wider discussion of the xenophobic assignment of blame for the pandemic since snakes feature saliently in the coverage of so-called “wet markets” as the potential source of the outbreak:

(9) Scientists who have been looking at the current coronavirus outbreak believe it comes from **snakes** and bats—animals that had been sold live at the Wuhan seafood market, before being killed and eaten (emphasis added, *Daily Mail Online*, 19th March, 2020).

A full exploration of this is beyond the scope of this paper, but naming strategies pertaining to “wet markets” are ideological as well. As Lin et al. (2021) discuss, many of the so-called “wet markets,” which are prevalent (not only) in east and southeast Asia, “sell only fresh produce and dead domesticated animals,” yet terminologically they “are often incorrectly conflated with live-animal or wildlife markets” (p. e386). Not only does this lack of differentiation potentially lead to a blanket-stigmatization of assumed “foreign” foodways (i.e., “alimentary xenophobia;” Chuvileva et al., 2020), the homogenization of all “wet markets” also makes it harder to create and implement policies targeting the relatively few which pose “a disproportionately large risk” (Lin et al., 2021, p. e392). The corpus does contain examples of this terminological conflation, also in broadsheets:

(10) “All the evidence gathered to date suggests that the now notorious Chinese “wet markets”—places selling live and dead animals for human consumption—provide an opportunity for coronaviruses to jump easily from animals to people.” (*The Guardian*, 25th March, 2020).

Therefore, it would be interesting to analyze terms used to refer to the Huanan Seafood Wholesale Market in Wuhan (which is a “wet market, live-animal market, and wildlife market”; Lin et al., 2021, p. e386) in particular but also lexical choices around “wet markets” in general and to explore whether the UK press has a tendency to construe “wet markets” as “universally dangerous instead of recognizing specific practices within them as predictable catalysts for preventable disease” (Chuvileva et al., 2020, p. 1).

## Summary and conclusion

This study has analyzed the distribution of “neutral” vs. “inappropriate” lexical choices in early UK newspaper coverage of the COVID-19 pandemic, focusing on terms used for the disease and the virus causing it. Overall, about 9% of all terms are “inappropriate,” with a stronger prevalence in “pre-naming” vs. “post-naming” and in tabloids vs. broadsheets. Furthermore, terms inciting undue fear and those containing geographic locations are particularly prevalent in terms of relative frequency. A closer look at the discourse context for selected terms (“killer bug,” “Wuhan (corona)virus,” Chinese (corona)virus' and “snake flu”) revealed that broadsheets tend to explicitly distance themselves from these terms, unambiguously evaluating them negatively (particularly “post-naming”), while tabloids tend to not problematize naming choices and also distance themselves from a negative evaluation of “inappropriate” terms by attributing the evaluation to someone else.

There are still multiple aspects of the rich dataset that were not explored here—apart from the naming choices around “wet markets” briefly discussed above, this includes the dispersion of terms (e.g., within individual articles or newspaper sections), semantic prosody, the analysis of images, or a closer analysis of “inappropriate” terms such as “killer virus” and how they are embedded in other “fear-inducing” language often found predominantly in tabloids (see, e.g., Wahl-Jorgensen, 2020). Lastly, it would be interesting to analyze articles explicitly covering Sinophobic and Anti-Asian incidents and hate crimes in terms of their construal in tabloids vs. broadsheets.

## Data availability statement

The original contributions presented in the study are included in the article/Supplementary material, further inquiries can be directed to the corresponding author.

## Author contributions

UK conducted all analyses presented here and was the only one involved in the composition of the manuscript.

## Conflict of interest

The author declares that the research was conducted in the absence of any commercial or financial relationships that could be construed as a potential conflict of interest.

## Publisher's note

All claims expressed in this article are solely those of the authors and do not necessarily represent those of their affiliated organizations, or those of the publisher, the editors and the reviewers. Any product that may be evaluated in this article, or claim that may be made by its manufacturer, is not guaranteed or endorsed by the publisher.

## Footnotes

^1^Even after the explicit discussion on COVID-19 nomenclature, problematic naming practices around pathogens and diseases persist, as evident in the discourse around the recent “monkeypox” outbreak (Roberts, 2022).

^2^While particularly the early phase of the COVID-19 pandemic is linked to a rise in Anti-Asian racism, it should be noted that later developments are associated with other forms of xenophobia, connected to the emergence and naming of different variants of the virus. Prominent examples are terms such as “UK/Kent,” “South African” or “Indian” variant. This is why the WHO introduced a new system for naming variants in May 2021. The use of letters of the Greek alphabet (e.g., Delta instead of Indian Variant) was suggested, complementing (though not replacing) the scientific names (such as B. 1.617. 2) in order “to simplify discussions but also to help remove some stigma from the names” (BBC, 2021).

^3^While the specificity of the dataset should be kept in mind and may be seen as a limitation, it should be noted that China is usually mentioned as the country with the first reported cases, often linked to the Huanan Seafood Wholesale Market. Furthermore, given the history of Anti-Asian prejudice outlined above, lexical choices around the disease as well as the virus and its potential origin are of particular interest in this context. This dataset thus allows for the identification of trends in the early reporting on COVID-19 in tabloid vs. broadsheet newspapers regarding these specific “politics of naming”.

^4^In a lot of cases expressions belong to more than one category—e.g., “deadly Chinese coronavirus” contains both a term inciting undue fear and a geographic location, so has been included in counts for both categories.
