# Supplementary material for: “Snake flu,” “killer bug,” and “Chinese virus”: A corpus-assisted critical discourse analysis of lexical choices in early UK press coverage of the COVID-19 pandemic
Source: Front Artif Intell. 2022 Nov 22;5:970972. doi: 10.3389/frai.2022.970972 (PMC9723132; doi:10.3389/frai.2022.970972)
Supplement: Supplementary file 1 [file Table_1.docx]

**Supplementary Table 1:** Exhaustive list of terms used to refer to COVID-19 and SARS-CoV-2 in the dataset, split into ‘pre-‘/and ‘post-naming’ for both broadsheet and tabloid newspapers. For each head noun, terms categorised as ‘neutral’ are listed first, followed by terms deemed ‘inappropriate’ in italics (the categorisation is based on the WHO guidelines). The number in brackets indicates how many of these instances were found in headlines. Overall counts for all ‘neutral’ and ‘inappropriate’ uses of each head noun as well as the overall ‘total’ for each head noun are included in bold. At the very bottom of the table, totals for ‘neutral’ vs. ‘inappropriate’ instances of all head nouns combined as well as the overall total are included.

|  | **Broadsheet**  **01/01-10/02** | **Broadsheet**  **11/02-31/03** | **Tabloid**  **01/01-10/02** | **Tabloid**  **11/02-31/03** | **Total** |
| --- | --- | --- | --- | --- | --- |
| The/a/- virus | 332 (4) | 459 (4) | 1707 (4) | 706 (8) | **3204 (20)** |
| Respiratory virus | - | - | 4 (-) | - | **4 (-)** |
| Fast-moving respiratory virus | 1 (-) | - | - | - | **1 (-)** |
| Latest virus | 1 (-) | - (-) | - (-) | - | **1 (-)** |
| Flu-like virus | 1 (-) | - | 3 (-) | 1 (-) | **5 (-)** |
| New respiratory virus | 1 (-) | - | - | - | **1 (-)** |
| Respiratory virus | - | 1 (-) | 4 (-) | - | **5 (-)** |
| Highly-contagious virus | 2 (-) | - | 4 (-) | 3 (-) | **9 (-)** |
| Highly infectious virus | - | 2 (-) | - | 1 (-) | **3 (-)** |
| New virus | 44 (-) | 6 (-) | 94 (1) | 21 (-) | **165 (-)** |
| Novel virus | 1 (-) | 4 (-) | 1 (-) | - | **6 (-)** |
| Never-before-seen virus | - | - | 13 (-) | 2 (-) | **15 (-)** |
| RNA virus | - | - | 16 (-) | - | **16 (-)** |
| Unnamed virus | - | - | 2 (-) | - | **2 (-)** |
| *Previously unknown virus* | *-* | *-* | *1 (-)* | *1 (-)* | ***2 (-)*** |
| *Deadly flu-like virus* | *-* | *-* | *2 (1)* | *-* | ***2 (1)*** |
| *Mysterious flu-like virus* | *-* | *-* | *1 (-)* | *-* | ***2 (-)*** |
| *Mysterious Sars-like virus* | *1 (-)* | *-* | *-* | *-* | ***1 (-)*** |
| *SARS-like virus* | *-* | *1 (-)* | *22 (3)* | *-* | ***23 (3)*** |
| *Deadly SARS-like virus* | *-* | *-* | *11 (-)* | *-* | ***11 (-)*** |
| *New SARS-virus* | *1 (1)* | *-* | *-* | *-* | ***1 (1)*** |
| *Deadly new respiratory virus* | *1 (-)* | *-* | *-* | *-* | ***1 (-)*** |
| *New and mysterious virus* | *1 (-)* | *-* | *-* | *-* | ***1 (-)*** |
| *Foreign virus* | *-* | *1 (-)* | *-* | *-* | ***1 (-)*** |
| *Deadly new virus* | *1 (-)* | *-* | *21 (-)* | *2 (-)* | ***24 (-)*** |
| *Mysterious virus* | *-* | *-* | *1 (-)* | *-* | ***1 (-)*** |
| *Mysterious new virus* | *1 (-)* | *-* | *3 (-)* | *-* | ***4 (-)*** |
| *Life-threatening virus* | *-* | *-* | *1 (-)* | *-* | ***1 (-)*** |
| *Deadly virus* | *16 (1)* | *5 (-)* | *79 (8)* | *46 (1)* | ***146 (-)*** |
| *New deadly virus* | *-* | *-* | *11 (-)* | *-* | ***11 (-)*** |
| *Killer virus* | *6 (-)* | *-* | *51 (5)* | *8 (-)* | ***65 (5)*** |
| *New killer virus* | *-* | *-* | *4 (-)* | *-* | ***4 (-)*** |
| *Mystery virus* | *2 (-)* | *-* | *14 (2)* | *1 (-)* | ***17 (3)*** |
| *Wuhan virus* | *18 (-)* | *-* | *10 (-)* | *3 (-)* | ***31 (-)*** |
| *Killer Wuhan virus* | *-* | *-* | *2 (-)* | *-* | ***2 (-)*** |
| *New Wuhan virus* | *-* | *-* | *2 (-)* | *-* | ***2 (-)*** |
| *China virus* | *2 (1)* | *1 (-)* | *7 (2)* | *-* | ***10 (3)*** |
| *Deadly china virus* | *-* | *-* | *2 (1)* | *-* | ***2 (1)*** |
| *Chinese virus* | *2 (-)* | *23 (3)* | *-* | *4 (-)* | ***29 (3)*** |
| *New Chinese virus* | *-* | *-* | *4 (-)* | *-* | ***4 (-)*** |
| *Novel Chinese virus* | *-* | *-* | *1 (-)* | *-* | ***1 (-)*** |
| *Mystery Chinese virus* | *1 (1)* | *-* | *-* | *-* | ***1 (1)*** |
| *Deadly mystery Chinese virus* | *-* | *-* | *1 (1)* | *-* | ***1 (1)*** |
| *Deadly Chinese virus* | *1 (1)* | *-* | *2 (-)* | *-* | ***3 (1)*** |
| *Wild bat virus* | *-* | *1 (-)* | *-* | *-* | ***1 (-)*** |
| *Bat virus* | *-* | *-* | *1 (-)* | *-* | ***1 (-)*** |
| *Snake virus* | *-* | *-* | *2 (1)* | *-* | ***2 (1)*** |
| **Total virus ‘neutral’** | **383 (4)** | **931 (4)** | **1848 (5)** | **734 (8)** | **3896 (21)** |
| ***Total virus ‘inappropriate’*** | ***54 (4)*** | ***32 (3)*** | ***258 (24)*** | ***65 (1)*** | ***409 (32)*** |
| **Total virus** | **437 (8)** | **963 (7)** | **2106 (29)** | **799 (9)** | **4305 (53)** |
|  |  |  |  |  |  |
| a/the/- coronavirus | 458 (35) | 667 (59) | 1391 (91) | 1172 (82) | **3688 (267)** |
| Corona | - | - | - | 13 (1) | **13 (1)** |
| Unnamed coronavirus | - | - | 9 (-) | - | **9 (-)** |
| Similar coronavirus | 2 (-) | - (-) | - | - | **2 (-)** |
| New coronavirus | 65 (-) | 55 (-) | 113 (1) | 66 (-) | **299 (1)** |
| Novel coronavirus | 35 (-) | 11 (-) | 73 (1) | 33 (-) | **152 (1)** |
| 2019 novel coronavirus | 2 (-) | - | - | 5 (-) | **7 (-)** |
| Latest strain of coronavirus | - | 1 (-) | - | - | **1 (-)** |
| Never-before-seen strain (of coronavirus) | - | - | 3 (-) | - | **3 (-)** |
| New strain (of) (the) coronavirus | 10 (-) | - (-) | 30 (-) | - | **40 (-)** |
| Another strain of coronavirus | - | - | - | 1 (-) | **1 (-)** |
| (New) form of coronavirus | 2 (-) | - | 2 (-) | - | **4 (-)** |
| New type of coronavirus | 1 (-) | - | 29 (-) | - | **30** |
| Previously unknown (type of) coronavirus | 3 (-) | - | - | - | **3 (-)** |
| Newly(-)identified coronavirus | 3 (-) | - | 3 (-) | - | **6 (-)** |
| Human coronavirus | 2 (-) | - | - | - | **2 (-)** |
| 2019-nCoV coronavirus | - | - | - | 9 (-) | **9 (-)** |
| COVID-19 coronavirus | - | 3 (-) | - | 3 (-) | **6 (-)** |
| severe acute respiratory syndrome coronavirus | - | 2 (-) | - | 2 (-) | **4 (-)** |
| *SARS-like coronavirus* | *-* | *-* | *13 (-)* | *-* | ***13 (-)*** |
| *Deadly new coronavirus* | *-* | *-* | *13 (-)* | *1 (-)* | ***14 (-)*** |
| *Mystery*  *new coronavirus* | *1 (-)* | *-* | *-* | *-* | ***1 (-)*** |
| *Deadly novel coronavirus* | *-* | *-* | *-* | *1 (-)* | ***1 (-)*** |
| *Deadly coronavirus* | *13 (3)* | *3 (-)* | *95 (4)* | *19 (-)* | ***130 (7)*** |
| *New (strain of) deadly coronavirus* | *-* | *-* | *4 (-)* | *-* | ***4 (-)*** |
| *China coronavirus* | *3 (3)* | *- (-)* | *8 (4)* | *-* | ***11 (7)*** |
| *Chinese coronavirus* | *-* | *-* | *29 (2)* | *-* | ***29 (2)*** |
| *New Chinese coronavirus* | *- (-)* | *1 (-)* | *7 (-)* | *-* | ***8 (-)*** |
| *New mysterious Chinese coronavirus* | *-* | *-* | *1 (-)* | *-* | ***1 (-)*** |
| *Deadly Chinese coronavirus* | *-* | *-* | *12 (1)* | *1 (-)* | ***13 (1)*** |
| *Killer Chinese coronavirus* | *-* | *-* | *4 (1)* | *-* | ***4 (1)*** |
| *China’s coronavirus* | *1 (-)* | *- (-)* | *-* | *-* | ***1 (-)*** |
| *Wuhan coronavirus* | *31 (1)* | *- (-)* | *124 (-)* | *2 (-)* | ***157 (1)*** |
| *Deadly Wuhan coronavirus* | *-* | *-* | *6 (-)* | *2 (-)* | ***8 (-)*** |
| *Killer Wuhan coronavirus* | *-* | *-* | *2 (-)* | *-* | ***2 (-)*** |
| *Latest Wuhan coronavirus* | *-* | *-* | *2 (-)* | *-* | ***2 (-)*** |
| *New Wuhan coronavirus* | *-* | *-* | *2 (-)* | *-* | ***2 (-)*** |
| *Wuhan novel coronavirus* | *3 (-)* | *- (-)* | *11 (-)* | *-* | ***14 (-)*** |
| *China’s deadly coronavirus* | *-* | *-* | *6 (1)* | *-* | ***6 (1)*** |
| *Lethal Wuhan strain (of coronavirus)* | *-* | *-* | *-* | *2 (-)* | ***2 (-)*** |
| **Total coronavirus ‘neutral’** | **673 (35)** | **739 (59)** | **1653 (93)** | **1302 (83)** | **4367 (270)** |
| ***Total* coronavirus *‘inappropriate’*** | ***52 (7)*** | ***4 (-)*** | ***339 (13)*** | ***28 (-)*** | ***423 (20)*** |
| **Total coronavirus** | **725 (42)** | **743 (59)** | **1992 (106)** | **1330 (83)** | **4790 (290)** |
|  |  |  |  |  |  |
| bug | - | 2 (-) | 12 (-) | 12 (1) | **26 (1)** |
| COVID-19 bug | - | - | - | 1 (-) | **1 (-)** |
| Super bug | - | 1 (-) | - | - | **1 (-)** |
| Vicious bug | - | - | 1 (-) | - | **1 (-)** |
| Highly-contagious bug | - | - | - | 1 (-) | **1 (-)** |
| Highly-infectious bug | - | - | 1 (-) | - | **1 (-)** |
| Pneumonia-causing bug | 1 (-) | - | - | - | **1 (-)** |
| *SARS-like bug* | *-* | *-* | *1 (-)* | *-* | ***1 (-)*** |
| *Life-threatening bug* | *-* | *-* | *-* | *1 (-)* | ***1 (-)*** |
| *Killer bug* | *-* | *-* | *8 (6)* | *9 (2)* | ***17 (8)*** |
| *Lethal bug* | *-* | *-* | *3 (-)* | *-* | ***3 (-)*** |
| *Deadly bug* | *-* | *-* | *-* | *4 (-)* | ***4 (-)*** |
| *Killer COVID-19 bug* | *-* | *-* | *-* | *2 (-)* | ***2 (-)*** |
| *Deadly flu-like bug* | *-* | *-* | *1 (-)* |  | ***1 (-)*** |
| *Mystery bug* | *-* | *-* | *1 (-)* | *-* | ***1 (-)*** |
| *Devil bug* | *-* | *-* | *1 (1)* | *-* | ***1 (-)*** |
| **Total bug ‘neutral’** | **1 (1)** | **3 (-)** | **14 (-)** | **14 (1)** | **32 (2)** |
| ***Total bug ‘inappropriate’*** | ***-*** | ***-*** | ***15 (6)*** | ***16 (2)*** | ***31 (8)*** |
| **Total bug** | **1 (1)** | **3 (-)** | **29 (6)** | **30 (3)** | **63 (10)** |
|  |  |  |  |  |  |
| nCoV-2019 | 4 (-) | - | - | - | **4 (-)** |
| 2019-nCoV | 19 (-) | 1 (-) | 82 (-) | 9 (-) | **111 (-)** |
| **Total nCoV ‘neutral’** | **23 (-)** | **1 (-)** | **82 (-)** | **9 (-)** | **115 (-)** |
| ***Total nCoV ‘inappropriate’*** | ***-*** | ***-*** | ***-*** | ***-*** | ***-*** |
| **Total nCoV** | **23 (-)** | **1 (-)** | **82 (-)** | **9 (-)** | **115 (-)** |
|  |  |  |  |  |  |
| SARS-CoV-2 | - | 6 (-) | - | 10 (-) | **16 (-)** |
| *Killer SARS-CoV-2 virus* | *-* | *-* | *-* | *1 (-)* | ***1 (-)*** |
| **Total SARS-CoV-2 ‘neutral’** | **-** | **6 (-)** | **-** | **10 (-)** | **16 (-)** |
| ***Total SARS-CoV-2 ‘inappropriate’*** | ***-*** | ***-*** | ***-*** | ***1 (-)*** | ***1 (-)*** |
| **Total SARS-CoV-2** | **-** | **6 (-)** | **-** | **11 (-)** | **17 (-)** |
|  |  |  |  |  |  |
| COVID/Covid | - | - | - | 5 (-) | **5 (-)** |
| Virus Responsible for COVID-19 | - | - | - | - | **0 (-)** |
| COVID-19 virus | - | 6 (-) | - | 9 (-) | **15 (-)** |
| COVID-19  (as virus) | - | 8 (1) | - | 34 (-) | **42 (1)** |
| Virus covid-19 | - | 1 (-) | - | 1 (-) | **2 (-)** |
| COVID-19  As illness | - | 204 (4) | - | 223 (-) | **427 (4)** |
| *Deadly COVID-19 virus* | *-* | *-* | *-* | *3 (-)* | ***(-)*** |
| **Total COVID-19 ‘neutral’** | **-** | **219 (5)** | **-** | **272 (-)** | **491 (5)** |
| ***Total COVID-19 ‘inappropriate’*** | ***-*** | ***-*** | ***-*** | ***3 (-)*** | ***2 (-)*** |
| **Total COVID-19** | **-** | **219 (5)** | **-** | **275 (-)** | **494 (5)** |
|  |  |  |  |  |  |
| Condition | 1 (-) | - | 12 (-) | - | **13 (-)** |
| Respiratory condition | - | - | 2 (-) | - | **2 (-)** |
| *Mystery condition* | *-* | *-* | *2 (-)* | *-* | ***2 (-)*** |
| *SARS-like condition* | *-* | *-* | *2 (-)* | *-* | ***2 (-)*** |
| *Killer condition* | *-* | *-* | *3 (-)* | *-* | ***3 (-)*** |
| **Total condition ‘neutral’** | **1 (-)** | **-** | **14 (-)** | **-** | **15 (-)** |
| ***Total condition ‘inappropriate’*** | ***-*** | ***-*** | ***7 (-)*** | ***-*** | ***7 (-)*** |
| **Total condition** | **1 (-)** | **-** | **21 (-)** | **-** | **22 (-)** |
|  |  |  |  |  |  |
| Flu | 2 (2) | - | - | - | **2 (-)** |
| *Wuhan flu* | *2 (-)* | *-* | *-* | *-* | ***2 (-)*** |
| *Wu flu* | *2 (-)* | *-* | *-* | *-* | ***2 (-)*** |
| *Snake flu* | *3 (-)* | *3 (-)* | *21 (1)* | *6 (-)* | ***33 (-)*** |
| *Serpent flu* | *2 (-)* | *-* | *-* | *-* | ***2 (-)*** |
| *Deadly snake flu* | *-* | *-* | *4 (-)* | *-* | ***4 (-)*** |
| *Deadly fish flu* | *-* | *-* | *2 (2)* | *-* | ***2 (-)*** |
| *Deadly Chinese snake flu* | *-* | *-* | *2 (-)* | *-* | ***2 (-)*** |
| **Total flu ‘neutral’** | **2 (2)** | **- (-)** | **- (-)** | **-** | **2 (-)** |
| ***Total flu ‘inappropriate’*** | ***7 (-)*** | ***3 (-)*** | ***29 (1)*** | ***6 (-)*** | ***45 (1)*** |
| **Total flu** | **9 (2)** | **3 (-)** | **29 (1)** | **6 (-)** | **47 (3)** |
|  |  |  |  |  |  |
| *Plague* | *-* | *1 (-)* | *1 (1)* | *1 (-)* | ***3 (2)*** |
| *New plague* | *-* | *2 (-)* | *-* | *-* | ***2 (-)*** |
| *Mystery plague* | *-* | *-* | *2 (1)* | *-* | ***2 (1)*** |
| *Chinese plague* | *-* |  | *-* | *1 (-)* | ***1 (-)*** |
| **Total plague ‘neutral’** | **-** | **-** | **-** | **-** | **-** |
| ***Total plague ‘inappropriate’*** | ***- (-)*** | ***3 (-)*** | ***3 (2)*** | ***2 (-)*** | ***8 (2)*** |
| **Total plague** | **- (-)** | **3 (-)** | **3 (2)** | **2 (-)** | **8 (2)** |
|  |  |  |  |  |  |
| Infection | 74 (1) | 47 (-) | 109 (-) | 122 (-) | **352 (1)** |
| Never-before-seen infection | - | - | 2 (-) | - | **2 (-)** |
| Highly contagious infection | - | - | 2 (-) | - | **2 (-)** |
| Extremely contagious infection | - | - | 11 (-) |  | **11 (-)** |
| Viral infection | 1 (-) | - | 1 (-) | - | **2 (-)** |
| Unnamed infection | - | - | 4 (-) | - | **4 (-)** |
| 2019-nCov infection | 1 (-) | - | - | 5 (-) | **6 (-)** |
| COVID-19 infection | - | 1 (-) | - | 3 (-) | **4 (-)** |
| COVID-19 infection(s) | - | 6 (-) | - | 1 (-) | **7 (-)** |
| Coronavirus infection | 2 (-) | 3 (1) | 6 (1) | 9 (-) | **20 (2)** |
| New coronavirus infection | 1 (-) | - | - | - | **1 (-)** |
| Novel coronavirus infection | - | - | 3 (-) | - | **3 (-)** |
| Respiratory infection | - | - | 1 (-) | - | **1 (-)** |
| Unnamed coronavirus infection | - | - | 1 (-) | - | **1 (-)** |
| Unusual respiratory infection | 1 (-) | - |  |  | **1 (-)** |
| Previously unknown coronavirus infection | 1 (-) | - | - | - | **1 (-)** |
| *Unknown infection* | *1 (-)* | *-* | *-* | *-* | ***1 (-)*** |
| *Mystery infection* | *-* | *-* | *2 (-)* | *-* | ***2 (-)*** |
| *Mystery lung infection* | *2 (-)* | *-* | *-* | *-* | ***2 (-)*** |
| *Killer COVID-19 infection* | *-* | *-* | *-* | *1 (-)* | ***1 (-)*** |
| *Deadly COVID-19 infection* | *-* | *-* | *-* | *1 (-)* | ***1 (-)*** |
| *Killer infection* | *-* | *-* | *4 (1)* | *2 (-)* | ***6 (1)*** |
| *Deadly infection* | *1 (-)* | *-* | *6 (-)* | *3 (-)* | ***10 (-)*** |
| *Fatal infection* | *-* | *-* | *1 (-)* |  | ***1 (-)*** |
| *SARS-like infection* | *-* | *-* | *21 (1)* | *-* | ***21 (1)*** |
| *Killer SARS-like infection* | *-* | *-* | *7 (-)* | *-* | ***7 (-)*** |
| *Deadly SARS-like infection* | *-* | *-* | *1 (-)* | *-* | ***1 (-)*** |
| *Mysterious respiratory infection* | *1 (-)* | *-* |  |  | ***1 (-)*** |
| *Life-threatening infection* | *-* | *-* | *5 (-)* | *2 (-)* | ***7 (-)*** |
| **Total infection ‘neutral’** | **81 (1)** | **57 (1)** | **140 (1)** | **140 (-)** | **418 (3)** |
| ***Total infection ‘inappropriate’*** | ***5 (-)*** | ***- (-)*** | ***47 (1)*** | ***9 (-)*** | ***61 (1)*** |
| **Total infection** | **86 (1)** | **57 (1)** | **187 (2)** | **149 (-)** | **479 (4)** |
|  |  |  |  |  |  |
| Disease | 100 (-) | 80 (1) | 192 (2) | 89 (-) | **461 (3)** |
| Covid-19 disease | - | 4 (-) | - | - | **4 (-)** |
| Viral disease | - | 1 (-) | - | - | **1 (-)** |
| New disease | 4 (-) | - | 5 (-) | - | **9 (-)** |
| Zoonotic disease | - | 1 (-) | 2 (-) | - | **3 (-)** |
| New respiratory disease | 4 (-) | - | - | - | **4 (-)** |
| Pneumonia-like disease | - | - | 1 (-) | - | **1 (-)** |
| New coronavirus disease | - | 1 (-) | - | - | **1 (-)** |
| New/novel coronavirus disease | - | 1 (-) | - | - | **1 (-)** |
| Novel coronavirus disease | - | 1 (-) | - | - | **1 (-)** |
| Coronavirus disease | - | - |  | 3 (-) | **1 (-)** |
| Notifiable disease | - | - | 1 (-) | 1(-) | **2 (-)** |
| airborne disease | - | - | 2 (-) | - | **2 (-)** |
| *Mystery China disease* | *1 (1)* | *-* | *-* | *-* | ***1 (1)*** |
| *Mystery new disease* | *2 (-)* | *-* | *-* | *-* | ***2 (-)*** |
| *Life-threatening disease* | *-* | *-* | *1 (-)* | *-* | ***1 (-)*** |
| *Deadly/deathly disease* | *2 (-)* | *-* | *22 (3)* | *6 (-)* | ***30 (3)*** |
| *Lethal disease* | *-* | *-* | *1 (-)* | *-* | ***1 (-)*** |
| *Killer disease* | *-* | *-* | *3 (1)* | *1 (1)* | ***4 (1)*** |
| *New killer disease* | *-* | *-* | *1 (-)* | *-* | ***1 (-)*** |
| *Mystery disease* | *-* | *-* | *2 (-)* |  | ***2 (-)*** |
| *SARS-like disease* | *-* | *-* | *2 (-)* | *1 (-)* | ***3 (-)*** |
| *New and dangerous respiratory disease* | *1 (-)* | *-* | *-* | *-* | ***1 (-)*** |
| *Potentially deadly respiratory disease* | *-* | *-* | *-* | *1 (-)* | ***1 (-)*** |
| **Total disease ‘neutral’** | **108 (-)** | **89 (1)** | **203 (2)** | **93 (-)** | **493 (3)** |
| ***Total disease ‘inappropriate’*** | ***6 (1)*** | ***- (-)*** | ***32 (4)*** | ***9 (1)*** | ***47 (6)*** |
| **Total disease** | **114 (1)** | **89 (1)** | **235 (6)** | **102 (1)** | **540 (9)** |
|  |  |  |  |  |  |
| Illness | 21 (-) | 9 (-) | 83 (-) | 52 (-) | **165 (-)** |
| New illness | 2 (-) | 1 (-) | - | 2 (-) | **4 (-)** |
| New viral illness | 1 (-) | - | - | - | **1 (-)** |
| ‘new viral coronavirus illness’ | 1 (-) | - | - | - | **1 (-)** |
| Respiratory illness | - | - | 8 (-) |  | **8 (-)** |
| Severe respiratory illness | 1 (-) | - | - | - | **1 (-)** |
| Contagious respiratory illness | 1 (-) | - | - | - | **1 (-)** |
| Highly-contagious illness | - | - |  | 1 (-) | **1 (-)** |
| Fast-spreading illness | - | - | 1 (-) |  | **1 (-)** |
| Contagious illness | - | - | 7 (1) | - | **7 (1)** |
| Covid-19 illness | - | 2 (-) | - | 1 (-) | **3 (-)** |
| Pneumonia-causing illness | - | - | 1 (-) | - | **1 (-)** |
| Flu-like illness | - | 1 (-) | 1 (-) | - | **2 (-)** |
| Influenza-like illness | - | 1 (-) | - | - | **1 (-)** |
| Pneumonia-like illness | 1 (-) | - | 6 (-) | 3 (-) | **10 (-)** |
| New pneumonia-type illness | 4 (-) | - |  |  | **4 (-)** |
| *Killer illness* | *-* | *-* | *6 (-)* | *1 (-)* | ***7 (-)*** |
| *Mystery illness* | *5 (-)* | *-* | *5 (-)* | *-* | ***10 (-)*** |
| *Mystery new illness* | *1 (-)* | *-* | *-* | *-* | ***1 (-)*** |
| *Deadly new illness* | *-* | *-* | *-* | *1 (-)* | ***1 (-)*** |
| *Deadly illness* | *1 (-)* | *1 (-)* | *2 (-)* | *1 (1)* | ***5 (1)*** |
| *Mysterious respiratory illness* | *1 (-)* | *-* | *-* | *-* | ***1 (-)*** |
| *SARS-like illness* | *2 (2)* | *-* | *1 (-)* | *-* | ***3 (2)*** |
| **Total illness ‘neutral’** | **32 (-)** | **14 (-)** | **107 (1)** | **59 (-)** | **212 (1)** |
| ***Total illness ‘inappropriate’*** | **14 (2)** | **1 (-)** | **14 (-)** | **3 (1)** | **32 (3)** |
| **Total illness** | **46 (2)** | **15 (-)** | **121 (1)** | **62 (1)** | **244 (4)** |
|  |  |  |  |  |  |
| Pneumonia | 22 (-) | 9 (-) | 213 (2) | 4 (-) | **248 (2)** |
| 2019-nCoV-induced pneumonia | - | - | 4 (-) | - | **4 (-)** |
| Viral pneumonia | 10 (1) | - | 7 (-) | - | **17 (1)** |
| Novel coronavirus pneumonia | - | - | - | 1 (-) | **1 (-)** |
| Never-before-seen pneumonia | 1 (-) | - | - | - | **1 (-)** |
| *Unexplained pneumonia* | *4 (-)* | *-* | *3 (-)* | *-* | ***7 (-)*** |
| *Unknown pneumonia* | *1 (-)* | *-* | *-* | *-* | ***1 (-)*** |
| *Life-threatening pneumonia* | *-* | *1 (-)* | *-* | *-* | ***1 (-)*** |
| *Wuhan pneumonia* | *2 (1)* | *-* | *2 (-)* | *-* | ***4 (1)*** |
| **Total pneumonia ‘neutral’** | **33 (1)** | **9 (-)** | **224 (2)** | **4 (-)** | **270 (3)** |
| ***Total pneumonia ‘inappropriate’*** | ***8 (1)*** | ***1 (-)*** | ***3 (-)*** | ***1 (-)*** | ***13 (1)*** |
| **Total pneumonia** | **41 (2)** | **10 (-)** | **227 (2)** | **5 (-)** | **283 (4)** |
|  |  |  |  |  |  |
| **Overall total ‘neutral’** | **1337 (44)** | **2068 (70)** | **4285 (104)** | **2637 (92)** | **10327 (310)** |
| **Overall total ‘inappropriate’** | **146 (15)** | **44 (3)** | **747 (51)** | **143 (5)** | **1080 (74)** |
| **Overall total** | **1483 (59)** | **2112 (73)** | **5032 (155)** | **2780 (97)** | **11407 (384)** |
