# Supplementary material for: “Snake flu,” “killer bug,” and “Chinese virus”: A corpus-assisted critical discourse analysis of lexical choices in early UK press coverage of the COVID-19 pandemic
Source: Front Artif Intell. 2022 Nov 22;5:970972. doi: 10.3389/frai.2022.970972 (PMC9723132; doi:10.3389/frai.2022.970972)
Supplement: Supplementary file 2 [file Table_2.docx]

**Supplementary Table 2:** Top 20 collocates of all instances of ‘coronavirus’ per sub-corpus (i.e., broadsheet pre-naming, broadsheet post-naming, tabloid pre-naming, tabloid post-naming; window: -3L, minimum collocate frequency: 5, MI + Log-Likelihood (p < 0.05), sorted by statistics). Collocates functioning as pre-modifiers/determiners in italics.

|  | **pre-naming** | **post-naming** |
| --- | --- | --- |
| **broadsheet** | *novel*, ongoing, examining, *strain*, due, treat, amid, *new*, *deadly*, contain, tested, toll, cases, affected, case, against, illness, over, identified, spread | *novel*, kill, wake, amid, coverage, combat, related, positive, treatment, fears, *deadly*, linked, tested, latest, impact, *new*, potential, response, against, spread |
| **tabloid** | exists, corresponded, reproduce, *novel*, corpses, version, sufferers, *type*, cured, carrying, associated, *killer*, related, *deadly*, itself, *strain*, genes, *unnamed*, caused, catch | *novel*, origins, cluster, amid, contracting, contract, catching, block, carry, wake, kill, largest, diagnosed, *2019-nCoV*, linked, positive, suspected, cure, *killer*, due |
