# Supplementary material for: “Snake flu,” “killer bug,” and “Chinese virus”: A corpus-assisted critical discourse analysis of lexical choices in early UK press coverage of the COVID-19 pandemic
Source: Front Artif Intell. 2022 Nov 22;5:970972. doi: 10.3389/frai.2022.970972 (PMC9723132; doi:10.3389/frai.2022.970972)
Supplement: Supplementary file 3 [file Table_3.docx]

**Supplementary Table 3:** Top 20 collocates of all instances of ‘virus’ per sub-corpus (i.e., broadsheet pre-naming, broadsheet post-naming, tabloid pre-naming, tabloid post-naming; window: -3L, minimum collocate frequency: 5, MI + Log-Likelihood (p < 0.05), sorted by statistics). Collocates functioning as pre-modifiers/determiners in italics.

|  | **pre-naming** | **post-naming** |
| --- | --- | --- |
| **broadsheet** | *killer*, investigate, contracted, contracting, suggesting, contagious, *deadly*, suggests, pass, died, test, believe, tested, *new*, get, spreading, where, case, about, *the* | fight, *deadly*, contain, diagnosed, positive, died, transmission, spread, infected *SARS(-CoV)*, impact, against, *Chinese*, cases, *the*, about, because, where, *this*, *COVID-19* |
| **tabloid** | mutations, ancestor, versions, believing, understanding, evolution, talking, RNA, posed, contracting, reveal, *mystery*, ability, positive, caught, immunity, exposed, contracted, *killer*, does | plausible, catch, *deadly*, *HIV*, exposure, catching, caught, contracting, contain, *highly*, *killer*, cure, suspected, potentially, negative, fight, carrying, toll, themselves, spreading |
